# Knowledge as “wings” and resilience as “armor”: a study on the impact of civil aviation pilots’ personal knowledge management abilities on career resilience

**DOI:** 10.3389/fpsyg.2025.1672381

**Published:** 2025-10-29

**Authors:** Fan Wu, Mimi Lai, Mingyang Li

**Affiliations:** ^1^School of Public Policy And Management, Guangxi University, Nanning, China; ^2^School of Public Administration, University of Electronic Science and Technology of China, Chengdu, China

**Keywords:** resilience, civil aviation safety, personal knowledge management ability, civil aviation pilots, career resilience

## Abstract

**Background:**

Pilots bear the critical responsibility of ensuring the safe operation of civil aviation, and their level of occupational resilience directly impacts the sustainable development of civil aviation safety. Based on this, this study is grounded in the global context of civil aviation safety development and draws on Conservation of Resources Theory and social exchange theory to construct a theoretical model of how individual knowledge management capabilities influence the occupational resilience of civil aviation pilots.

**Methods:**

The respondents in this study were recruited using random sampling methods, with questionnaires distributed online and offline between January and June 2025. The survey mainly covered pilots from China’s four major airline groups. Participants were asked to complete the Positive Personal Knowledge Management Ability Scale, Career Resilience Scale, Safety Behavior Scale, Safety Climate Scale, and Work Pressure Scale. A total of 210 valid questionnaire data from Chinese civil aviation pilots were collected.

**Results:**

First, personal knowledge management ability significantly positively affects the career resilience of civil aviation pilots (β = 0.654, *p* < 0.001), and safety behavior plays a partial mediating role in the relationship between the two. Second, safety climate (β = −0.028, *p* < 0.05) and work stress (β = −0.026, *p* < 0.05) both showed an inverted U-shaped moderating effect on the influence of personal knowledge management ability on the career resilience of civil aviation pilots. Third, heterogeneity analysis found that the personal knowledge management ability of pilots from key universities (β = 0.773, *p* < 0.001) and domestic aviation colleges (β = 0.707, *p* < 0.001) had a stronger promoting effect on career resilience.

**Conclusion:**

The research results clarify the process mechanism by which personal knowledge management ability stimulates the career resilience of civil aviation pilots, enriches the study of personal career resilience, and provides theoretical and practical inspiration for ensuring civil aviation flight safety.

## 1 Introduction

Amid the dual trends of sustained expansion in global civil aviation and deep integration with digital transformation, aviation safety has risen to become a critical issue concerning social stability and national sustainable development. Currently, the aviation safety system faces the combined impact of traditional safety risks and non-traditional security challenges. The former stems from airspace resource constraints caused by increased flight density and heightened uncertainties in the flight environment. Even though the 2023 safety report released by the International Civil Aviation Organization (ICAO) indicates that the global aviation accident rate has dropped to 1.87 per million flights, and China’s commercial air transport sector leads globally with a major accident rate of 0.0249 per million flights over a 10-year rolling period. Nevertheless, the complexity of traditional risks—such as the sudden nature of aircraft mechanical failures and the pressure to maintain punctuality under high-density operations—continues to intensify. The latter challenge is driven by digital transformation. The intelligent evolution of flight equipment and the refinement of safety management not only demand that practitioners master new skills like data interaction and system fault diagnosis, but also require the conversion of fragmented regulatory provisions and special situation handling cases into systematic knowledge. Moreover, the dynamic updating of emergency response plans further shortens the knowledge lifecycle. Amid these dual challenges, the career stability and adaptability of civil aviation professionals no longer depend solely on skill proficiency. Instead, they hinge on the ability to respond to risks and the agility to iterate knowledge. As the direct executors of flight operations and the core guardians of safety, pilots’ resilience in facing pressure and challenges directly impacts both their individual career prospects and the foundational safety of the civil aviation industry (Miani et al., 2021; Yiu et al., 2022).

Against this backdrop, personal knowledge management ability emerges as a critical lever for pilots to navigate dual challenges and fortify career resilience. On one hand, systematically cataloging incident response experiences and operational deviation cases transforms tacit knowledge into structured resources, enhancing efficiency in handling unexpected events within traditional safety scenarios. On the other hand, proactively integrating digital skills and dynamically updating emergency knowledge builds personalized knowledge repositories tailored to non-traditional safety requirements, preventing skill obsolescence. More importantly, the knowledge confidence cultivated through this management process effectively alleviates career anxiety and combats work burnout, providing dual psychological and competency support for career resilience. Existing research on career resilience has made some progress in terms of connotation (London, 1983), structural models (London and Noe, 1997), measurement dimensions (Grzeda and Prince, 1997), influencing factors and outcomes (Fisher and Stafford, 2000), but the scope of research has mostly focused on general workplace environments. In the high-risk and highly specialized field of civil aviation, research on the mechanisms influencing career resilience, especially among pilots, remains weak. In terms of personal knowledge management ability, existing research mainly focuses on definitions (Chuang, 2004), measurement dimensions (Gold et al., 2001), and the impact on corporate or individual innovation performance (Bao et al., 2024). Although previous studies have made many useful explorations, and some studies have confirmed that knowledge management capabilities have a positive impact on individual performance, in the special field of civil aviation, the mechanism linking it to career resilience is not yet clear, and the transmission effect of safety behavior between the two has not been addressed. At the same time, the role of boundary conditions has been neglected, and there has been a particular lack of discussion of the nonlinear effects of variables such as safety climate and work pressure in the “personal knowledge management ability—safety behavior—career resilience” chain. Based on this, this study is based on the global civil aviation safety development context, with Chinese civil aviation pilots as the research object, integrates Conservation of Resources Theory and Social Exchange theory, and attempts to construct a mediating model of “personal knowledge management ability—safety behavior—career resilience,” introducing safety climate and work pressure as moderating variables to systematically examine the boundary effects of the two in the above-mentioned transmission process. The aim is to reveal the interaction between the mediating mechanism of safety behavior and the two moderating variables, fill the gap in research on the synergistic effects of multiple variables on career resilience in high-risk occupational contexts, and provide a targeted theoretical basis and practical path for the synergistic optimization of “personal knowledge management ability,” “safety behavior norms,” and “resilience systems” in the training of Chinese civil aviation pilots.

The theoretical and practical significance of this study manifests in three key aspects: First, it addresses gaps in research on high-risk occupational settings and expands the boundaries of career resilience studies. By extending resilience research from general workplace contexts to high-risk scenarios in civil aviation, this study fills a research void in high-risk professions, providing new application scenarios and empirical support for career resilience theory. Second, it unravels the black box of mechanisms among variables, refining the influence pathway of personal knowledge management ability on career resilience. By constructing an ‘individual knowledge management ability—safety behavior—career resilience’ mediation model, this study systematically validates the mediating role of safety behavior. It clearly deconstructs the complete pathway through which personal knowledge management ability influences career resilience, thereby enriching the research content on the impact mechanism between these two constructs. Third, it uncovers nonlinear relationships among variables, challenging linear moderation effect conclusions. Based on Conservation of Resources Theory and the Yerkes-Dodson law, this study innovatively proposes and verifies an inverted U-shaped moderating effect of safety climate and work pressure on the pathway from personal knowledge management ability to career resilience. This provides a more practical theoretical explanation for understanding the synergistic mechanisms of multiple variables in high-risk occupational settings. It also offers actionable practical pathways for civil aviation enterprises to optimize pilot training systems and enhance pilot career resilience.

## 2 Theoretical foundation and research hypotheses

### 2.1 Personal knowledge management ability and career resilience of civil aviation pilots

The term “resilience” was first applied in the field of psychology, primarily emphasizing how individuals cope with difficulties and adversity (Ma and Tang, 2024). Career resilience is a core concept in the fields of organizational behavior and career development, and its theoretical connotations have continued to evolve and enrich with further research. Early studies defined it as “an individual’s resistance to career disruption in a pessimistic environment” (London, 1983) and as a professional quality (Youssef and Luthans, 2007). it enables individuals to quickly bounce back or recover from adversity, conflict, and failure, as well as from positive events, progress, and increasing responsibilities (Luthans et al., 2006), and in this recovery process, they achieve a higher cognitive level (Xu and Ling, 2007). Career resilience is a positive factor that runs through the process of environmental change (Caverley, 2005) and not only effectively eliminates career plateau dilemmas (Zhi and Ren, 2023), but also helps to improve work performance (He et al., 2023), job satisfaction, and career identity (Zhang and Cui, 2023). In the civil aviation field, career resilience is of great significance for preventing and reducing pilot burnout and ensuring aviation safety (Li et al., 2023). In terms of influencing factors, the formation and development of career resilience is driven by multiple variables: at the demographic level, age (Brainerd, 1992; Fisher and Stafford, 2000; Noe et al., 1990), gender (London and Noe, 1997), and educational level (Lin, 2009; Liu, 2003) significantly influence its development. At the personal trait level, optimism, internal control, and positive coping styles (Caverley, 2005), self-discipline, creativity, endurance, perseverance (Grzeda and Prince, 1997), and self-efficacy (Fisher and Stafford, 2000) also have a positive impact on an individual’s career resilience. Scholars have mainly discussed the connotation, measurement dimensions, influencing factors, and results of career resilience, but there is still room for expansion in the field of civil aviation safety development.

Knowledge resources are a new type of production factor that replaces labor and capital, including intangible assets such as human capital and information technology (Jia and Yang, 2009); knowledge management capability refers to the ability to create, organize, transfer, and utilize knowledge resources, as well as the ability to integrate knowledge resources with other resources and capabilities to promote value creation (Chuang, 2004), which is divided into knowledge infrastructure capability and process capability (Gold et al., 2001). As the environment changes rapidly, organizational resilience requires human resource management strategies to support the development of individual knowledge, skills, and capabilities through organizational learning (Douglas and Haley, 2024). Knowledge production is not only a driving factor for organizational resilience but also determines its complexity and effectiveness (Blyth et al., 2025). Knowledge management, measured by knowledge acquisition, knowledge storage, knowledge sharing, and knowledge utilization, has a significant impact on the business resilience of micro-entrepreneurs (Zayed et al., 2022). Additionally, knowledge management capabilities positively promote the growth of small and medium-sized enterprises from both scale expansion and qualitative optimization perspectives and can significantly positively influence the construction of organizational resilience (Zheng et al., 2022). Four key areas of human resource practices—job design, information sharing and flow within the organization, employee benefits, and employee development opportunities—can foster employee resilience (Khan et al., 2019). In terms of research on knowledge management infrastructure capabilities and innovative work behaviors, knowledge management infrastructure capabilities have a significant positive impact on personal resilience (Nassani et al., 2024).

From the perspective of complex adaptive systems theory, individuals function as dynamic adaptive systems that continuously adjust their internal structures through ongoing interaction with the environment to maintain system resilience. At its core lies the “learning-adaptation-evolution” feedback loop: Knowledge acquisition serves as the information interface between the system and the flight environment, injecting initial resources into the system. Knowledge storage forms the system’s “memory module” through structured encoding, while knowledge application represents the system’s output response to environmental changes. During this process, the cognitive system rapidly retrieves relevant knowledge to adjust strategies and maintain stability, with the emergence of stability constituting the core of cognitive resilience. Additionally, the results of knowledge application drive knowledge updates through feedback loops, continuously enhancing the system’s adaptability to similar changes. This enhances the consistency of behavioral responses when pilots repeatedly face high-pressure situations, thereby improving behavioral resilience. In addition, the system actively reserves “redundant knowledge” through personal knowledge management ability, providing a buffer space for emergency response and reducing the risk of system collapse. This cognition alleviates emotional tension and ultimately acts to enhance emotional resilience, forming a path of resilience enhancement through dynamic adaptation between “individual-knowledge-environment.” In summary, this study proposes Hypothesis 1:

H1: Personal knowledge management ability has a significant positive impact on the career resilience of civil aviation pilots.

H1a: Personal knowledge management ability has a significant positive impact on the affection resilience of civil aviation pilots.

H1b: Personal knowledge management ability has a significant positive impact on the behavior resilience of civil aviation pilots.

H1c: Personal knowledge management ability has a significant positive impact on the cognition resilience of civil aviation pilots.

### 2.2 The mediating effect of safety behavior

Safety is the lifeline of the civil aviation industry, and pilots, as the primary responsible parties for ensuring the safety of flight operations, play a critical role in the safe operation of aircraft. Numerous career development factors result in pilots experiencing significantly higher work-related stress compared to other professions, and such stress can influence individual behavior (Brown and Holmes, 1986). Work-related stress can significantly increase the likelihood of unsafe behaviors (Zhou et al., 2022). Accumulating safety knowledge can effectively enhance safe behaviors, thereby reducing workplace accidents and occupational hazards (Li et al., 2025) and minimizing stress and anxiety caused by unexpected events or unsafe factors. When employees continuously accumulate safety knowledge and perceive the safety of their work environment, they develop enhanced psychological safety, enabling them to confidently address occupational challenges and difficulties (Xu and Peng, 2017).

Safety itself is a resource, and safety resources encompass those resources available for safety and related to safety (Wu and Wang, 2024). The Conservation of Resources Theory holds that individuals tend to accumulate, protect, and maintain their own resources, enhance their adaptability and psychological security, promote investment and development of existing resources, and form positive psychological states and behavioral performance. When individuals face stress, they will prioritize the protection of existing resources and strive to acquire new resources to cope with challenges, thereby achieving continuous growth and improving career resilience (Näswall et al., 2015). First, personal knowledge management ability provides the “source of motivation” for resource accumulation. The core function of personal knowledge management ability is to help pilots accumulate safety-related “knowledge resources,” which are the basic reserves for individuals to cope with environmental challenges and provide cognitive support for subsequent behavior. Second, safe behavior is the “key carrier” for transforming knowledge resources into “protective effectiveness.” Knowledge resources alone are not enough to directly enhance career resilience; they need to be transformed into actual resource protection capabilities through safety behaviors. From the perspective of resource preservation, safety compliance behaviors are “defensive resource protection.” Pilots use knowledge resources to strictly implement standard operations and procedures, which can directly avoid resource losses caused by operational errors. Safety participation behaviors represent “proactive resource enhancement.” By sharing knowledge resources, individuals can gain team recognition while also acquiring new safety knowledge through interaction. These two types of behaviors collectively transform static knowledge resources into dynamic resource protection and enhancement capabilities, forming a conversion chain of “knowledge resources-behavioral output-resource homeostasis.” Finally, maintaining resource homeostasis will support the improvement of career resilience. The essence of career resilience is the ability of individuals to maintain resource homeostasis in adverse conditions. When pilots achieve resource protection and value-added through safe behavior, the sustained maintenance of this resource homeostasis ultimately manifests itself in improved career resilience—that is, they are less likely to be defeated by adversity and can recover quickly from setbacks. In summary, this study proposes Hypothesis 2:

H2: Safety behavior plays a mediating role in the impact of personal knowledge management ability on the career resilience of civil aviation pilots.

### 2.3 The moderating role of safety climate and work pressure

Safety climate refers to individuals’ perceptions of workplace safety-related policies, procedures, and practices (Neal and Griffin, 2006) and is influenced by both individual factors (such as personality and attitude) and situational factors (such as organizational climate, job requirements, job resources, and leadership) (He et al., 2019). In fact, safety climate may vary depending on individual perceptions (Zohar and Luria, 2005). Empowering leadership creates a favorable safety climate, empowering employees to participate in decision-making, encouraging them to solve problems and share knowledge together, and improving knowledge sharing behavior, willingness to participate in safety, and safety compliance (Lee et al., 2019), thereby improving individual career resilience. However, leadership style has a double-edged effect. Inclusive leadership has a contradictory impact on employees’ creativity (Zhu et al., 2020). It positively influences creativity through team psychological safety and subordinates’ motivational dependence, while subordinates’ cognitive dependence negatively impacts creativity (Gu et al., 2017). Inclusive leadership promotes subordinates’ deviant innovative behavior but may also lead to knowledge hiding behaviors that are detrimental to innovation (Ye et al., 2020).

Social exchange theory points out that the interaction between individuals and organizations is essentially a process of “resource exchange,” in which both sides maintain a balance of exchange through reciprocal investment, and this balance directly affects the attitudes and behavioral choices of individuals. As an “safety support signal” at the organizational level, differences in the intensity of the safety climate can change the pattern of resource exchange between pilots and organizations, thereby exerting a nonlinear regulatory effect on the relationship between “personal knowledge management ability and career resilience.” The specific mechanism is reflected in three stages. In the low safety climate stage, the safety resources provided by the organization, such as systematic safety training, a comprehensive emergency support system, and leadership attention to safety, are relatively scarce, and the signals that pilots receive about the organization’s emphasis on safety are vague. At this time, the exchange relationship between pilots and the organization exhibits “low reciprocity”-individuals perceive that the organization’s investment in safety is insufficient, reduce their dependence on organizational resources, and instead build a “self-protection resource pool” by strengthening their personal knowledge management abilities. At this point, the positive relationship between personal knowledge management ability and career resilience is amplified by “compensatory needs,” manifesting as a stage of increasing regulatory effects. In the moderate safety climate stage, the organization and pilots form a balanced exchange relationship, in which the organization provides basic safety resources, and pilots repay the organization’s investment through compliant behavior. The “reciprocity norm” in social exchange theory plays a central role at this stage-pilots perceive the organization’s safety support, reduce their over-reliance on personal knowledge management, and integrate organizational resources with personal knowledge resources. This collaborative model not only avoids resource redundancy in a low climate but also reduces idle capacity in a high climate, maximizing the role of personal knowledge management ability in improving career resilience and forming the apex of an inverted U-shaped curve. In the high safety climate stage, the safety resources provided by the organization are in a “saturated state”—including comprehensive monitoring systems, redundant emergency support processes, and overly detailed operating guidelines. At this point, the exchange relationship between pilots and the organization may show a tendency toward “excessive reciprocity,” that is, individuals gradually reduce their active investment in personal knowledge management due to long-term reliance on the organization’s “safety net” mechanism, leading to stagnant knowledge updates and a decline in emergency decision-making abilities. At this point, the role of personal knowledge management ability is squeezed by organizational resources, and its positive impact on career resilience is weakened, forming the downward phase of the inverted U-shaped curve. In summary, this study proposes hypothesis H3:

H3: The safety climate has an inverted U-shaped moderating effect on the impact of personal knowledge management ability on the career resilience of civil aviation pilots.

Work-related stress refers to the physical and mental tension individuals experience in the workplace. Excessive work-related stress can negatively impact and harm an individual’s physical and mental health, and have adverse effects on organizations (Shi, 2002). Stress does not necessarily have only negative effects (Wallace et al., 2009); individuals may distinguish between different types of stress based on their own interests and disadvantages. There is both a positive and negative correlation between stress and work outcomes. Self-reported stress related to challenges is positively correlated with job satisfaction and negatively correlated with job search; self-reported stress related to obstacles is negatively correlated with job satisfaction and positively correlated with job search and turnover (Cavanaugh et al., 2000). Stress related to challenges in the learning environment is positively correlated with learning performance, while stress related to obstacles in the learning environment is negatively correlated with learning performance (LePine et al., 2004).

The Yerkes-Dodson law reveals the nonlinear relationship between stress intensity and task performance. Moderate stress can activate an individual’s physiological and psychological arousal levels and improve task performance, while too much or too little stress can lead to a decline in performance. This law provides a theoretical basis for analyzing the moderating effect of work stress on the relationship between personal knowledge management ability and career resilience. During periods of low work stress, pilots’ cognitive arousal is at a basic level, and there is a large surplus of cognitive resources. According to the characteristics of the low arousal range of the Yerkes-Dodson law, pilots lay a solid cognitive foundation for career resilience through continuous expansion of their knowledge reserves. At this point, the positive impact of personal knowledge management ability on career resilience is reinforced by sufficient resources, forming the ascending phase of an inverted U-shaped curve. During periods of moderate work stress, pilots’ cognitive arousal is at the “optimal threshold” of the Yerkes-Dodson law-maintaining a high level of concentration without cognitive overload. In this state, the “application effectiveness” of personal knowledge management ability reaches its peak. At the same time, moderate stress also stimulates a positive “stress-growth” cycle, enhancing pilots’ recognition of the value of knowledge, thereby actively optimizing their knowledge structure and continuously improving their career resilience, forming the peak of the inverted “U” curve. During periods of high work stress, pilots’ cognitive arousal enters a state of over activation, manifested in narrowed attention, a sharp decrease in working memory capacity, and a decline in information processing speed. The exercise of personal knowledge management ability will also be limited, and this “knowledge management failure” directly weakens the foundation for the generation of career resilience. When pilots are unable to effectively call on knowledge resources to cope with adversity, their career resilience and adaptability will significantly decline, forming the downward phase of the inverted U-shaped curve. In summary, this study proposes hypothesis H4:

H4: Work pressure has an inverted U-shaped moderating effect on the impact of personal knowledge management ability on the career resilience of civil aviation pilots.

**Figure 1:**
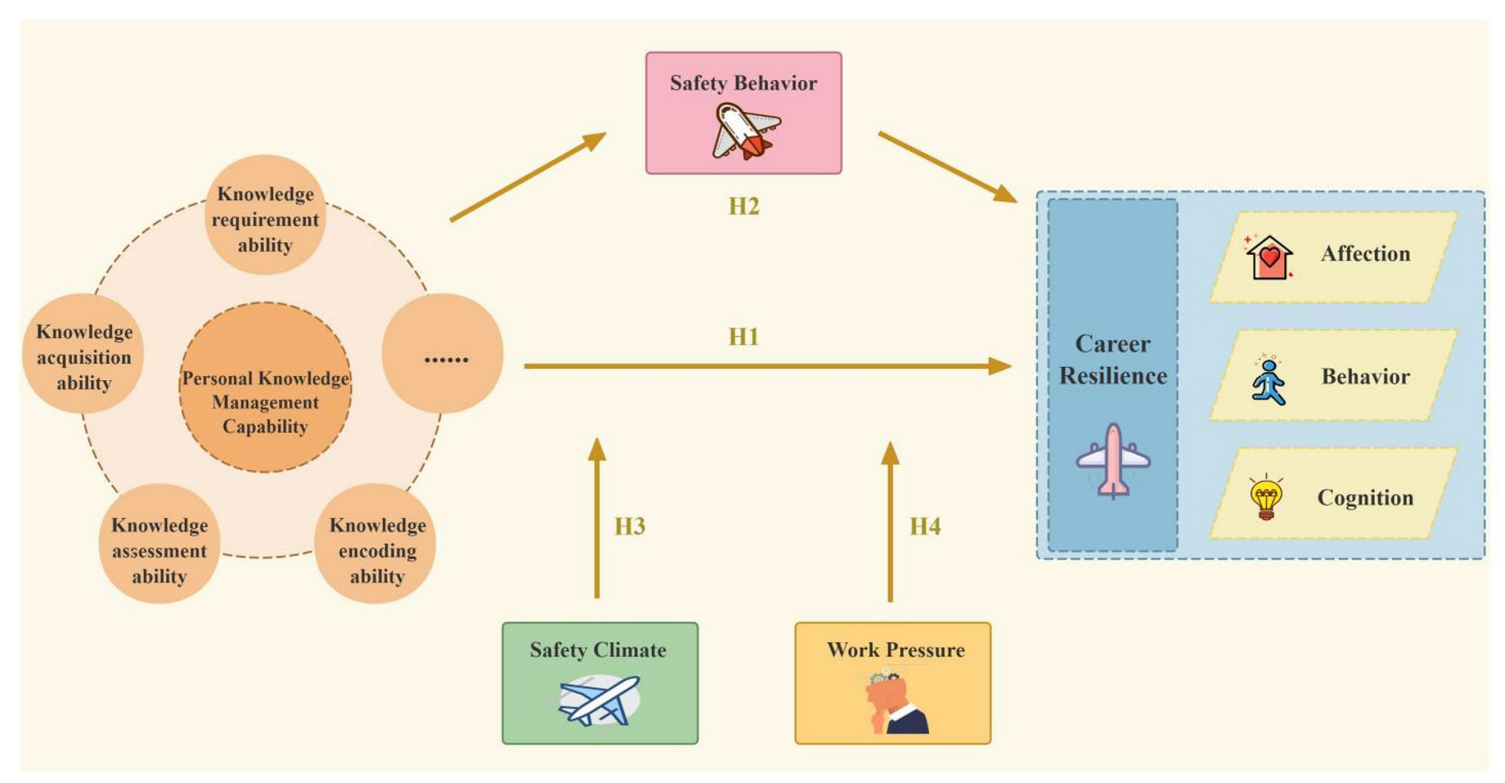
Theoretical framework.

The theoretical model of this study is shown in Figure 1.

## 3 Research design

### 3.1 Data collection and sample

Considering the availability of the data, this study focuses on Chinese civil aviation pilots^1^ as the research subjects. A questionnaire survey method was employed, with questionnaires distributed and collected through a combination of online and offline methods. The survey scope primarily included pilots from China’s four major aviation groups.^2^ A total of 250 questionnaires were distributed for this survey, and 220 were returned. After sorting the questionnaire data and removing invalid questionnaires, 210 valid questionnaires were obtained, with a validity rate of 84%.

The sample in this study exhibits distinct industry characteristics: first, in terms of age structure, pilots aged 20–30 account for a significantly higher proportion, which aligns with the trend toward a younger pilot workforce driven by the rapid development of China’s civil aviation industry in recent years; Second, in terms of education and background, since China’s civil aviation pilots generally follow an educational system combining “higher education at universities+specialized flight training at aviation schools,” the majority of practitioners hold a bachelor’s degree as their highest level of education. This also results in a relatively high proportion of pilots with flight training school experience in the sample.

The reasons for selecting Chinese civil aviation pilots as the sample group include: First, China is one of the world’s largest aviation markets. This market positioning necessitates that its pilots manage a more diverse range of flight scenarios. They must first cover busy domestic and international transport routes. Beyond this, they must also account for the unique characteristics of China’s airspace resource structure—addressing operational challenges arising from tight airspace resources and the coordination of multi-domain requirements, while simultaneously meeting the special operational requirements of complex plateau-terrain airports. Second, China’s civil aviation pilot training system exhibits distinct characteristics, combining foundational university education with specialized flight school training. Studying this cohort enables in-depth analysis of how this specific training model influences pilots’ knowledge management capabilities and career resilience, providing insights for optimizing global pilot training approaches. The statistical summary of the sample for this study is shown in Table 1.

**Table 1:** Summary of sample statistics.

Variable	Classify	Number
Gender	Male	210
	Female	0
Age	20–30 years	184
	31–40 years	20
	41–50 years	4
	51 years and above	2
Marital status	Unmarried	119
	Married, No Children	45
	Married, With Children	45
	Divorced	1
Career background^1^	University-trained Pilots	162
	Postgraduate Career Changers	33
	Pregraduate Career Changers	14
	Military-to-Civilian	1
Flight training school	Domestic Aviation Schools	150
	Foreign Aviation Schools	60
University category^2^	Key Universities	28
	Non-Key Universities	182
Highest educational attainment	Associate Degree or Below	0
	Bachelor’s Degree	208
	Master’s Degree or Above	2

1 There are four types of career backgrounds among Chinese pilots: “University-trained Pilots” refers to pilots who have received flight training from university and obtained a Commercial Pilot License (CPL). “Postgraduate Career Changers” refers to those with a university degree who later attended a flight school and obtained a CPL. “Pregraduate Career Changers” refers to individuals who switched from a non-aviation major to flight training before earning a CPL. “Military-to-Civilian “refers to former military pilots who transitioned into civil aviation.

2 Historically, Chinese universities have been categorized under different classifications such as “985,” “211,” and “Double First-Class” institutions. In this study, they are divided into two groups: “Key Universities,” which include those listed under “985,” “211,” and “Double First-Class” programs, comparable to Ivy League institutions; and “Non-Key Universities,” referring to those outside these classifications.

### 3.2 Variable measurement

All scales used in this study are well-established scales that have been widely used and proven to have good reliability and validity. Based on the actual research subjects, this study uses a 5-point Likert scale, where 1 to 5 represent “strongly agree,” “agree,” “unsure,” “disagree,” and “strongly disagree,” respectively.

Core explanatory variables: This study measures personal knowledge management ability using the mature scale developed by Zheng (2018), which mainly measures nine dimensions, including knowledge requirements, acquisition, and assessment ability. The index system for personal knowledge management ability is shown in Table 2. From a commonality perspective, the scale covers the entire process of knowledge management and is highly consistent with the entire chain of knowledge processing requirements of the pilot profession, including the internalization of initial knowledge, knowledge integration in crew collaboration, transfer and application in special situation handling, and flight procedure optimization. From the perspective of specificity, the pilot profession has extremely high requirements for the accuracy, timeliness, and safety of knowledge, and the nine dimensions of this scale can capture these characteristics separately. The scale has been empirically tested and has good reliability and validity, providing a standardized tool for measuring and improving the personal knowledge management ability of pilots, and is therefore suitable for related research.

**Table 2:** Indicator system of personal knowledge management ability.

Level 1 indicators	Level 2 indicators	Level 3 indicators
Personal knowledge management ability	Knowledge Requirement Ability	I have clear objectives for knowledge needs when acquiring knowledge
	Knowledge Acquisition Ability	I am able to use tools and software to acquire the knowledge I need
	Knowledge Assessment Ability	I am able to differentiate between the value of different knowledge and filter the knowledge.
	Knowledge Encoding Ability	I have a set of criteria for categorizing knowledge.
	Knowledge Storage Ability	I can categorize and store acquired knowledge in my personal knowledge base.
	Knowledge organization Ability	I often organize my knowledge to eliminate outdated or useless knowledge from my knowledge base.
	Knowledge Sharing Ability	I take the initiative to share my knowledge online and offline.
	Knowledge Application Ability	I often utilize theoretical knowledge to solve practical problems.
	Knowledge Innovation Ability	After acquiring knowledge, I am good at combining and internalizing it into new knowledge to achieve new understanding.

Core explanatory variable: Career resilience is measured using Song’s (2011) 25-item scale, which divides career resilience into three dimensions: affection, behavior, and cognition (Table 3). This scale is also well suited to measuring the career resilience of civil aviation pilots, mainly based on the following considerations: First, from the perspective of the scale’s inherent characteristics, it was developed based on the Chinese cultural context, emphasizing indigenous elements such as “collective support” (cooperative awareness) and “long-term development” (long-term orientation). Through localization research, the scale has been validated to have high structural validity, internal consistency reliability, and criterion-related validity, effectively capturing the core competencies individuals possess in coping with occupational stress and adversity. Second, in terms of the alignment between the occupational characteristics of pilots and the scale dimensions, the high-risk, high-stress, highly collaborative, and rapidly evolving technological environment of their profession aligns closely with the scale’s dimensions. For example, the behavioral dimension aligns with the continuous skill updates and career planning required by China’s civil aviation development, reflecting the industry’s practical demands for technological iteration and lifelong learning. Finally, the scale’s focus on “comprehensive qualities for coping with career stress” is highly consistent with the requirements for coping with the career stress faced by civil aviation pilots (such as safety responsibility stress and work-life balance stress). Therefore, the scale can be effectively used for measuring pilot career resilience and related research.

**Table 3:** Career resilience indicator system.

Level 1 Indicators	level 2 indicators	Level 3 Indicators	Level 4 indicators
Career Resilience (CR)	Affection Dimensions	Career Enthusiasm	Works with enthusiasm and overcomes problems
			Enjoys his/her career field and is not afraid of encountering problems.
			Enthusiastic to work even when faced with career pressures
		Sense of cooperation	Positively win the understanding, care and support of family members
			Actively seeks help from others when career difficulties arise
			Willingness to establish and maintain friendships with people in different departments
			Willingness to build up a network of relationships that can help career development
	Behavior Dimensions	Long-term orientation	Establishes long-term career development goals and is psychologically prepared for setbacks on the development path
			Explore the future direction of your career field to make timely adjustments
			Career dilemmas are only temporary when handled correctly
			Explore the changes that are occurring in your career field in order to adapt
		Willingness to learn	Enhance necessary competencies and skills to cope with difficult situations
			Participate in learning to acquire new knowledge to cope with stress
			Actively pursue learning opportunities to improve career skills
			Continuously improve skills to meet career needs
			Improvement from difficult situations
			Enhancement of competencies and skills necessary for career change
	Cognition Dimensions	Adaptability	Ability to adapt to work pressure
			Ability to plan for difficult situations
			Acceptance of work and organizational changes
			Ability to take career risks even when the outcome is uncertain
		Self-efficacy	Believes that he/she is able to deal with career dilemmas in an appropriate manner
			Believes that he/she has sufficient knowledge/skills to perform his/her job well
			Confident enough to overcome difficulties
			Able to control their unpleasant emotions

Mediating variables: Measurement of safety behavior draws on Neal’s (Neal and Griffin, 2006) 6-item scale, which includes two sub-dimensions: safety compliance and safety participation. The selection of Neal’s scale to measure the dual dimensions of safety compliance and safety participation among Chinese civil aviation pilots is fundamentally based on its ability to comprehensively capture the theoretical implications and practical characteristics of safety behavior in the high-risk civil aviation industry. Safety compliance focuses on procedural norm adherence, which serves as the foundational pillar of civil aviation safety management. Safety participation, on the other hand, reflects organizational citizenship behavior, embodying proactive safety contributions beyond basic duties. Together, these two dimensions form a continuous behavioral spectrum ranging from “passive compliance” to “proactive co-creation.” Sample items include: “I use all necessary safety equipment to complete my work,” “I use the correct safety procedures to perform my work,” and “I ensure the highest level of safety when performing my work.”

Moderating variables: Work stress and safety climate were selected as moderating variables in this study because they jointly construct a boundary condition system for the transformation of pilots’ knowledge management capabilities into career resilience, which can explain the different mechanisms of this transformation process in different organizational contexts and individual states. The measurement of work pressure and safety climate was based on the scales developed by Wang and Zhang (2016) and Neal and Griffin (2006), respectively. The measurement of work pressure consisted of three questions, such as “My work is extremely stressful” and “There are few things in my work that are not stressful.” The measurement of safety climate consisted of three questions, such as “Management attaches great importance to health and safety in the workplace” and “Management attaches great importance to safety.”

Control Variables: Based on previous research, age (AG), marital status (MS), highest educational attainment (HEA), and career background (CB) were included as control variables. Due to space limitations, abbreviations of variables are used in the following table.

### 3.3 Model selection

#### 3.3.1 Linear regression model

Drawing upon the research findings of scholars such as Cao and Wang (2025) and Shan et al. (2025), this study employs a linear regression model for analysis for the following reasons: First, the linear regression model demonstrates greater applicability. The logit/probit model is primarily suited for binary categorical dependent variables, whereas the core dependent variable in this study is a typical continuous variable. This aligns perfectly with the linear regression model, allowing direct modeling without additional data processing. This approach preserves complete data integrity while precisely quantifying the marginal effects of independent variables on the dependent variable (Freedman, 2009). Second, the linear regression model offers superior interpretability. It enables clear understanding of variable influence direction and magnitude without external tools. The model structure is fully transparent, with predictive logic fully expressed through mathematical formulas—eliminating black-box components. Predictions for any sample can be precisely decomposed into the contribution of the intercept and each variable, clearly identifying positive and negative influencing factors. More importantly, it allows verification of coefficient significance through statistical methods like t-tests and F-tests, calculation of confidence intervals, and direct estimation of the average causal effect of variables on the dependent variable when causal assumptions are satisfied (Efron and Hastie, 2021). Therefore, based on survey data from 210 Chinese civil aviation pilots, this study constructs an empirical model examining the impact of personal knowledge management ability on career resilience among civil aviation pilots. As shown in Equation 1:


(1) $$\mathrm{CR}=\theta_{1}+\text{cPKMA}+\kappa_{1}X+\varepsilon$$

In Equation 1, *“CR”* is the dependent variable, representing the career resilience level of Chinese civil aviation pilots;*“* $\;\theta_{1}$ *”* is the intercept term; *PKMA* is the explanatory variable, representing the personal knowledge management ability of civil aviation pilots; *“c”* is the estimated coefficient, representing that each unit increase in personal knowledge management ability leads to a *β_1_* unit change in the level of career resilience of pilots; “*k_1_”* is the estimated coefficient of the control variable; *“X”* is the control variable that affects the career resilience of pilots, including age, marital status, highest educational attainment, and growth experience;*“* ε *”* is the error term. The coefficient in Equation 1 can be used to determine the impact of personal knowledge management ability on the career resilience of pilots. If c > 0, it indicates that personal knowledge management ability helps to enhance the career resilience of civil aviation pilots; otherwise, it inhibits the career resilience of civil aviation pilots.

#### 3.3.2 Mediating effect model

In order to further understand the mechanism of personal knowledge management’s impact on the career resilience of civil aviation pilots, this study draws on the mediation effect testing method proposed by Baron and Kenny (1986), using safety behavior as a mediating variable to construct a mediation model based on the above equation, as shown in Equations 2 and 3:


(2) $$M=\theta_{2}+\text{αPKMA}+\kappa_{2}X+\varepsilon$$

(3) $$\mathrm{CR}=\theta_{3}+c\prime \text{PKMA}+\mathrm{bM}+\kappa_{3}X+\varepsilon$$

In Equations 2 and 3, *“M”* is the mediating variable, *“* α *”* is the estimated coefficient corresponding to *PKMA*, and represents the effect of personal knowledge management ability on the mediating variable; *“k_2_*, *k_3_”* is the control variable coefficient; *“c’”* is the estimated coefficient of *PKMA* after adding the mediating variable; and *“b”* is the estimated coefficient of the mediating variable.

#### 3.3.3 Moderating effect model

In order to further understand the nonlinear moderating role of safety climate and work stress in promoting career resilience among civil aviation pilots, this study constructed a nonlinear moderation effect model, as shown in Equation 4:


(4) $$\begin{array}{l} \mathrm{CR}=\beta_{0}+\beta_{1}\text{PKMA}+\beta_{2}Z+\beta_{3}Z^{2}+\beta_{4}(\text{PKMA}\times Z)\\ +\beta_{5}(\text{PKMA}\times Z^{2})+\kappa_{4}X+\varepsilon \end{array}$$

In Equation 4, *“Z”* is the moderating variable,*“* $\beta_{2}$ *”* is the estimated coefficient corresponding to the moderating variable, and *“* $\beta_{5}$ *”* is the estimated coefficient corresponding to the interaction term between the independent variable and the square of the moderator variable.

## 4 Results

### 4.1 Common method bias and scale reliability and validity testing

#### 4.1.1 Common method bias test

Since all research data were derived from self-reports by participants, potential homogeneity bias exists. To mitigate the impact of homogeneity bias on findings, this study implemented both pre-control measures and post-hoc verification. During the pre-control phase, the questionnaire explicitly stated confidentiality principles to participants and emphasized that the survey and collected data were solely for academic research purposes. For post-hoc verification, the study first employed Harman’s one-factor ANOVA. Results indicated that the first factor explained 8.477% of the variance, which is less than 40%. Then, Confirmatory Factor Analysis (CFA) was conducted by grouping all measurement items into a single factor. The measurement results showed that the model fit indices did not meet the criteria for good fit, indicating that all measurement items did not belong to the same latent factor. This ultimately confirmed that the common method bias issue was not significant.

#### 4.1.2 Reliability and validity testing

Due to the specific nature of the research subjects, the reliability and validity of the scales were first tested, with the following results.

First, the Cronbach’s α coefficients of the career resilience scale, personal knowledge management ability scale, and work pressure scale were all greater than 0.8; the Cronbach’s α coefficients of the safety behavior scale and safety climate scale were both greater than 0.7. The KMO value was 0.882, greater than 0.5, and Bartlett’s sphericity test significance level P was less than 0.001, indicating that the data test results were suitable for factor analysis.

Second, the composite reliability (CR) of career resilience, personal knowledge management ability, safety behavior, safety climate, and work stress scales ranged from 0.773 to 0.876, with CR greater than 0.7, indicating that the scales used in the study had good reliability. Aggregate validity was assessed using average variance extracted (AVE), and the results showed that the AVE values of career resilience, personal knowledge management ability, safety behavior, safety climate, and work pressure scales ranged from 0.365 to 0.585, all greater than the acceptable range of 0.36 (Yang et al., 2023), indicating that the scales used in this study have good convergent validity. Discrimination validity was tested using the HTMT method (heterogeneity-to-homogeneity ratio), and the ratio of inter-trait correlation to intra-trait correlation was less than 0.85, indicating that the career resilience, personal knowledge management ability, safety behavior, safety climate, and work pressure involved in this study can be distinguished from each other and are suitable for further analysis.

### 4.2 Descriptive statistics and correlation analysis

Before conducting regression analysis, descriptive statistics were calculated for each variable (Table 4). Using SPSS software, this study computed data including minimum, maximum, mean, and standard deviation values. For Career Resilience (CR), the minimum value was 1.00, maximum value was 3.00, mean was 1.85, and standard deviation was 0.45. The explanatory variable, Personal Knowledge Management Ability (PKMA), the minimum value was 1.00, maximum value was 3.22, mean was 1.93, and standard deviation was 0.52. Other variables are not detailed individually.

**Table 4:** Descriptive statistics of variables.

Variable	Sign	Minimum value	Maximum value	Mean value	Standard deviation	*N*
Career resilience	*CR*	1.00	3.00	1.85	0.45	210
Personal knowledge management ability	*PKMA*	1.00	3.22	1.93	0.52	210
Safety behavior	*SB*	1.00	4.33	1.85	0.54	210
Work pressure	*WP*	1.00	4.33	2.14	0.79	210
Safety climate	*SC*	1.00	5.00	1.73	0.66	210
Age	*AG*	1.00	5.00	1.17	0.51	210
Marital status	*MS*	1.00	4.00	1.66	0.83	210
Highest educational attainment	*HEA*	2.00	3.00	2.01	0.10	210
Career background	*CB*	1.00	4.00	1.30	0.61	210

**Figure 2:**
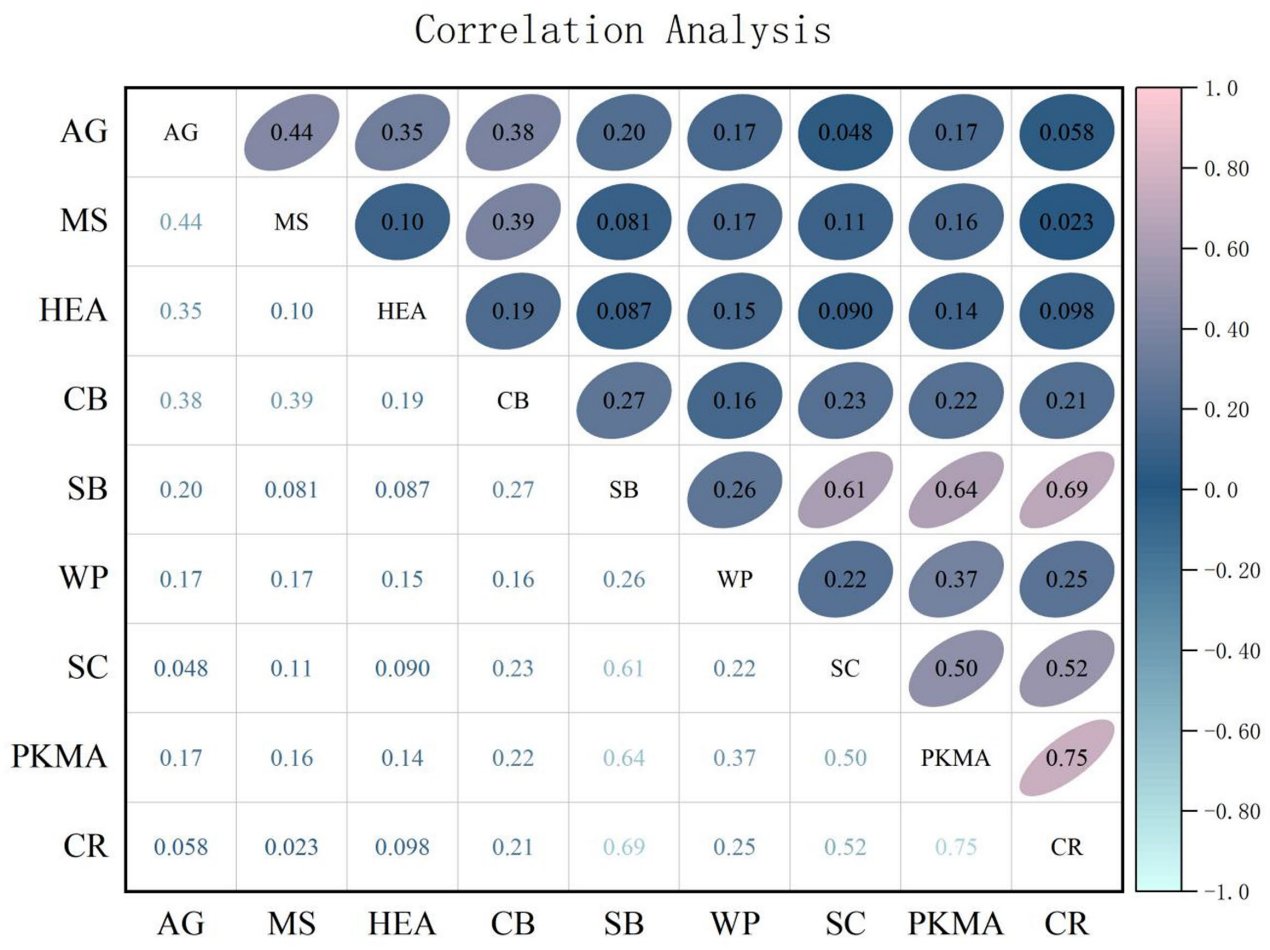
Correlation analysis.

The correlation analysis in Figure 2 indicates that the correlation coefficient between personal knowledge management ability and career resilience of civil aviation pilot is 0.75, which is significant at the 1% level. This suggests that personal knowledge management ability and pilot career resilience exhibit a certain degree of correlation. Simultaneously, correlations also exist between personal knowledge management ability and safety behavior, as well as between safety behavior and career resilience of civil aviation pilot. The correlation coefficients between different variables are all below 0.8, and the VIF values are well below 5. This indicates that there is no multicollinearity issue among the variables, making them suitable for empirical analysis.

### 4.3 Hypothesis testing

#### 4.3.1 Testing the relationship between personal knowledge management ability and career resilience

This study uses a regression method to examine the relationship between personal knowledge management ability and career resilience among civil aviation pilots. Model 1 in Table 5 shows that, under the condition of controlling variables, personal knowledge management ability has a significant positive effect on career resilience (β = 0.654, p<0.001), and hypothesis H1 is verified. This conclusion is highly consistent with the requirements and professional characteristics of China’s civil aviation industry. From the perspective of industry practice, on the one hand, in order to meet the pressure of technological iteration, the Chinese civil aviation industry is accelerating the promotion of evidence-based training (EBT) and pilot life cycle management (PLM), which requires pilots to shift from the traditional “technology-driven” paradigm to a three-dimensional “human-machine-environment” paradigm. Personal knowledge management ability can help pilots continuously update their skill maps through systematic knowledge acquisition and internalization, ensuring that they remain fit for their jobs amid changes such as aircraft model upgrades and process optimization, avoiding the devaluation of professional abilities caused by technological disconnect, and laying a solid skill foundation for career resilience. On the other hand, the phenomenon of a large number of flight trainees being forced to change careers due to delays in conversion during the epidemic highlights the structural contradiction between career development uncertainty and the backlog of trainees. Personal knowledge management ability gives pilots the ability to develop independently across organizational constraints. This knowledge-driven career navigation alleviates the risk of burnout caused by unclear career paths and allows them to maintain a stable mindset in the face of career fluctuations, which is of great practical significance for improving the level of civil aviation safety.

**Table 5:** Regression results for personal knowledge management ability and career resilience.

Variables	CR Model 1	Affection Model 2	Behavior Model 3	Cognition Model 4
*PKMA*	0.654*** (16.189)	0.661*** (12.180)	0.660*** (14.925)	0.641*** (12.091)
*AG*	−0.057 (−1.173)	−0.049 (−0.765)	−0.024 (−0.454)	−0.103 (−1.637)
*MS*	−0.064* (−2.249)	−0.043 (−1.112)	−0.069* (−2.209)	−0.077* (−2.060)
*HEA*	0.022 (0.098)	−0.060 (−0.199)	−0.195 (−0.790)	0.365 (1.236)
*CB*	0.085* (2.237)	0.085 (1.662)	0.103* (2.480)	0.062 (1.254)
*Constance*	0.608 (1.385)	0.676 (1.147)	0.962 (2.003)	0.106 (0.183)
*R* ^2^	0.586	0.445	0.549	0.443
∆*R*^2^	0.576	0.432	0.538	0.429
*F*-Value	57.740	32.731	49.719	32.431

*p < 0.05, **p < 0.01, ***p < 0.001. Values in parentheses represent *t*-values.

Furthermore, we examined the specific impact of personal knowledge management ability on career resilience from the three dimensions—affective, behavioral, and cognitive. Models 2, 3, and 4 in Table 6 show that personal knowledge management ability has a significant positive impact on the affective (β = 0.661, *p* < 0.001), behavioral (β = 0.660, *p* < 0.001), and cognitive (β = 0.641, *p* < 0.001) dimensions of career resilience. Hypothesis H1a, Hypothesis H1b, and Hypothesis H1c are validated. The core logic of complex adaptive systems theory posits that individuals, as dynamic adaptive systems, must continuously adjust their internal cognitive, behavioral, and affective structures through ongoing information exchange with the external environment to achieve a closed-loop cycle of “learning-adaptation-evolution.” This closed-loop mechanism is precisely the key process for maintaining and enhancing career resilience among civil aviation pilots. Given the realities of China’s civil aviation industry, pilots endure prolonged pressures from stringent regulatory constraints, complex airspace environments, and frequent technological upgrades, making their career resilience critically important. On the emotional level, strong personal knowledge management ability—such as systematically acquiring flight policies, aircraft model knowledge, meteorological information, and safety regulations—significantly enhances pilots’ sense of control over the flight environment and their confidence in competence. This not only effectively reduces career anxiety caused by ambiguous or delayed information but also maintains professional identity and emotional balance, ultimately enhancing affective resilience. At the behavioral level, uncertainties in China’s civil aviation operations—such as handling special situations, sudden flight changes, and introducing new aircraft types or technologies—place extreme demands on pilots’ behavioral stability and response efficiency. Efficient knowledge integration, retrieval, and application enable pilots to rapidly mobilize procedural knowledge and situational experience for effective responses, maintaining operational stability and mission execution capability—a direct manifestation of behavior resilience. At the cognitive level, China’s civil aviation industry is in a critical phase of consolidating safety foundations, enhancing development quality, and deepening comprehensive reforms. Pilots must continuously adapt themselves. Through personal knowledge management—involving critical reflection and systematic structuring of flight experiences, case reviews, and industry dynamics—pilots can transform abnormal events into learning opportunities, fostering positive cognitive attitudes that strengthen cognition resilience. Thus, personal knowledge management ability systematically enhances pilots’ comprehensive resilience in affect regulation, behavior resilience, and meaning construction by providing continuous knowledge support and cognitive restructuring resources. This holds significant practical importance for ensuring the operational safety of China’s civil aviation and maintaining the stability of its pilot workforce.

**Table 6:** Results of the mediating effect analysis of safety behavior.

Variables	CR		
	Model 1	Model 2	Model 3
*PKMA*	0.654*** (16.189)		0.463*** (9.874)
*SB*		0.582*** (13.324)	0.301*** (6.556)
*AG*	−0.057 (−1.173)	−0.112* (−2.089)	−0.092* (−2.080)
*MS*	−0.064* (−2.249)	−0.006 (−0.176)	−0.041 (−1.554)
*HEA*	0.022 (0.098)	0.325 (1.309)	0.111 (0.540)
*CB*	0.085* (2.237)	0.049 (1.159)	0.046 (1.322)
*Constance*	0.608 (1.385)	0.194 (0.397)	0.291 (0.724)
*R* ^2^	0.586	0.494	0.658
∆*R*^2^	0.576	0.482	0.648
*F*-Value	57.740	39.864	65.181

*p < 0.05, **p < 0.01, ***p < 0.001. Values in parentheses represent t-values.

#### 4.3.2 Testing the mediating effect of safe behavior

This study used the mediation effect test method proposed by Baron and Kenny (1986). As can be seen from Models 1, 2, and 3 in Table 6, when personal knowledge management ability and safety behavior simultaneously predict career resilience, the regression coefficients of personal knowledge management ability (β = 0.463, *p* < 0.001) and safe behavior (β = 0.301, *p* < 0.001) have significant regression coefficients for career resilience, but the regression coefficient of personal knowledge management ability for career resilience decreases from 0.654 to 0.463, indicating that safe behavior partially mediates the effect of personal knowledge management ability on the career resilience of civil aviation pilots, and hypothesis H2 is verified.

Further analysis of the mediating effect of safety behaviors reveals that its partial mediation clearly demonstrates the transmission pathway of ‘personal knowledge management ability—safety behaviors—career resilience.’ This pathway not only resonates deeply with Conservation of Resources Theory but also precisely aligns with civil aviation’s dual-track safety requirements of ‘manual execution + situational judgment,’ fully illustrating the theoretical logic of resource accumulation—behavioral transformation—resilience enhancement in the civil aviation context. From the Conservation of Resources Theory, the core of individual resilience lies in maintaining and enhancing resource homeostasis. The mediating role of safety behaviors essentially transforms safety knowledge resources accumulated through personal knowledge management ability into a resource homeostasis that underpins career resilience—via two behavioral pathways: safety compliance and safety participation. This process aligns closely with the practical requirements of civil aviation safety operations and can be explored through these two dimensions. in terms of safety compliance, civil aviation pilots use their personal knowledge management abilities to update their professional knowledge, operating procedures, and flight skills in a timely manner, and then transform that knowledge into standardized behavior to reduce flight risks caused by unknown deviations. This standardized operation forms the foundation of career resilience. At the level of safety participation, personal knowledge management ability encourages pilots to actively share their experience in handling special situations and participate in safety training optimization. Through knowledge interaction, they enrich the experience reserve required for situational judgment and form the driving force for career resilience. Based on this, the impact of personal knowledge management ability on career resilience directly improves pilots’ emergency response capabilities and resistance to career disruption when faced with unknown risks through knowledge updating and learning. It also indirectly strengthens the impact of personal knowledge management ability on career resilience through safety compliance and safety participation, which fully meets the multiple safety requirements of the civil aviation industry.

Finally, this study further examined the mediating effect of safety behavior using Preacher and Hayes’ (2008) Bootstrap mediation effect test method. The Bootstrap sample size was set to 5,000, with a 95% confidence interval. If the 95% confidence interval did not include 0, it indicated the presence of a mediating effect; if the 95% confidence interval included 0, it indicated the absence of a mediating effect. The results are shown in Table 7. The mediating effect of safety behavior is 0.198, with a 95% confidence interval of [0.153, 0.328], which does not include 0, indicating that the mediating effect of safety behavior is significant. After controlling for the mediating variable, the direct effect of personal knowledge management ability on career resilience is still significant (direct effect = 0.452***, *p* < 0.001), and the direct effect value of 0.452 is less than the total effect value of 0.651, indicating that safety behavior plays a partial mediating role. The hypothesis is further verified.

**Table 7:** Bootstrap test results.

Path	Effect value	2SE	95% Confidence interval		
			Lower	Upper	*p*
*PKMA→SB→CR*	0.198	0.044	0.153	0.328	0.000***

*p < 0.05, **p < 0.01, ***p < 0.001.

#### 4.3.3 Testing the moderating effects of safety climate and work pressure

This study first examines the moderating role of safety climate in the impact of personal knowledge management ability on the career resilience of civil aviation pilots. After centralizing the data, the results show that the interaction term between personal knowledge management ability and safety climate squared is significantly negative (β = −0.028, *p* < 0.05). The results are shown in Table 8, indicating that there is a nonlinear inverted “U” relationship between the strength of the safety climate and career resilience. That is, the safety climate may overemphasize rules and procedures, limiting the freedom of individuals to acquire and utilize knowledge, or causing individuals to become overly dependent on the safety framework provided by the organization, thereby weakening or offsetting the positive impact of personal knowledge management ability on career resilience, or even having a reverse impact on career resilience. Hypothesis H3 is validated.

**Table 8:** Moderating effect analysis of safety climate.

Variables	Model 1	Model 2	Model 3
PKMA *SC^2^			−0.028* (−2.530)
PKMA*SC			−0.147** (−2.873)
*SC^2^*		0.019* (2.573)	0.033*** (3.610)
SC		0.132*** (3.762)	0.174*** (4.650)
*PKMA*	0.661*** (15.391)	0.620*** (13.642)	0.583*** (12.407)
*AG*	−0.056 (−1.152)	−0.042 (−0.880)	−0.034 (−0.719)
*MS*	−0.063* (−2.198)	−0.068* (−2.390)	−0.068* (−2.419)
*HEA*	0.031 (0.135)	0.010 (0.045)	0.065 (0.292)
*CB*	0.085* (2.242)	0.075* (2.000)	0.065 (1.740)
*Constant*	1.877*** (4.248)	1.930*** (4.423)	1.846*** (4.276)
*R* ^2^	0.586	0.600	0.612
∆*R*^2^	0.586	0.013	0.012
*F*-Value	47.981	43.210	39.621

*p < 0.05, **p < 0.01, ***p < 0.001. Values in parentheses represent *t*-values.

The core logic of social exchange theory lies in maintaining a dynamic equilibrium of resource exchange between individuals and organizations. The inverted U-shaped moderating effect of safety climate on “personal knowledge management ability—career resilience among civil aviation pilots” fundamentally stems from shifts in the “safety resource exchange patterns between pilots and organizations” under varying safety climate intensities. These shifts subsequently influence the effectiveness of knowledge management abilities in transforming into career resilience. In a safety climate of appropriate intensity, civil aviation pilots can sense the importance that the organization and other crew members place on safe flying from their surroundings. They will then consciously or unconsciously be influenced by the environment, take the initiative to learn professional knowledge and skills, and turn this into a habit. This strong atmosphere of knowledge sharing and learning gives pilots more courage and ability to actively respond to risks and challenges when faced with unexpected situations, thereby enhancing their career resilience. When the safety climate exceeds a certain level, pilots are prone to “free riding,” that is, when all organizations and individuals focus on flight safety and improving professional knowledge and skills, individuals are prone to neglect safety inspections and their own professional knowledge and skills, believing that the organization and team members will solve all safety issues for them, thereby weakening their own enthusiasm and initiative. At the same time, when the entire organization overemphasizes the safety climate, it may increase the frequency of safety inspections, equip too much safety equipment, and add too many safety inspection procedures, which not only takes up other resources, but also makes pilots feel tired and stressed, thereby weakening and even negatively affecting their career resilience. This mechanism aligns with the core logic of social exchange theory while precisely addressing the industry’s reality of balancing aviation safety and efficiency, providing theoretical support for understanding the nonlinear regulatory role of safety climate.

Second, we examined the moderating effect of work stress on the impact of personal knowledge management ability on the career resilience of civil aviation pilots. The interaction term between personal knowledge management ability and work pressure squared was also significantly negative (β = −0.026, *p* < 0.05). The results are shown in Table 9, indicating that there is a nonlinear inverted “U” relationship between work stress and career resilience, that is, moderate work stress can stimulate personal knowledge management ability and further promote the development of career resilience; however, when stress exceeds a certain threshold, excessive work stress may prevent individuals from effectively utilizing knowledge resources, thereby weakening career resilience. Hypothesis H4 is validated.

**Table 9:** Moderating effect analysis of work pressure.

Variables	Model 1	Model 2	Model 3
PKMA* WP^2^			−0.026* (−2.354)
PKMA* WP			−0.121** (−2.617)
*WP^2^*		−0.007 (−1.159)	−0.005 (−0.848)
WP		−0.018 (−0.673)	−0.019 (−0.702)
*PKMA*	0.576*** (12.963)	0.590*** (12.824)	0.565*** (12.108)
*SC*	0.131*** (3.742)	0.131*** (3.740)	0.135*** (3.899)
*AG*	−0.038 (−0.809)	−0.035 (−0.755)	−0.020 (−0.429)
*MS*	−0.066* (−2.397)	−0.064* (−2.311)	−0.066* (−2.404)
*HEA*	−0.006 (−0.026)	0.017 (0.078)	0.080 (0.364)
*CB*	0.064 (1.711)	0.064 (1.725)	0.060 (1.636)
*Constant*	1.706*** (3.973)	1.653*** (3.831)	1.526*** (3.550)
*R^2^*	0.613	0.615	0.626
∆*R*^2^	0.613	0.003	0.010
*F-Value*	53.518	46.142	41.975

*p < 0.05, **p < 0.01, ***p < 0.001. Values in parentheses represent *t*-values.

A possible explanation is that the core mechanism of work stress lies in dynamically changing the effectiveness boundary of the transformation of knowledge resources into resilience through two paths: the efficiency of cognitive resource allocation and the threshold of motivational activation. Under a certain intensity of work pressure, such as time pressure (ensuring flight punctuality), safety pressure (regulations and standard operating procedures), physiological pressure (fatigue, hypoxia, and noise effects), and weather/environmental pressure (wind shear, thunderstorm avoidance, low visibility, and strong turbulence), pilots can be stimulated to engage in “challenging evaluations,” placing them in the optimal arousal state described by the Yerkes-Dodson Law. Under excessive work pressure, pilots are prone to emotional states such as tension and anxiety, which can affect their decision-making quality and problem-solving abilities. Even with a certain amount of knowledge reserves, it is difficult to effectively use this knowledge to make correct decisions under high pressure, leading to a decline in career resilience. At the same time, prolonged high pressure can cause pilots to feel physically and mentally exhausted, making it difficult to concentrate on effective personal knowledge management, which in turn weakens the positive impact of personal knowledge management ability on career resilience.

Therefore, work pressure is not merely an external disturbance variable but is deeply embedded within the psychological processes of knowledge activation and resilience development. Its effects follow the nonlinear patterns revealed by the Yerkes-Dodson Law. This underscores the need for civil aviation management practices to prioritize the identification and regulation of stress levels. This involves providing task challenges sufficient to maintain optimal arousal levels while simultaneously preventing excessive stress accumulation through organizational support, resource allocation, and mental health interventions—thus averting cognitive and motivational resource depletion.

### 4.4 Heterogeneity analysis

The samples were divided into key universities and non-key universities, and the regression results are shown in Model 1 and Model 2 in Table 10. It can be observed that, compared with non-key universities, the personal knowledge management ability of pilots from key universities has a stronger promoting effect on career resilience. The reason may be as follows: key universities are generally considered to be high-quality resources in China’s higher education system, with significant advantages in terms of teaching staff, teaching facilities, scientific research levels, and academic reputation. In contrast, non-key universities may have certain deficiencies in these areas. According to Conservation of Resources Theory, key universities leverage their superior educational resources to help pilots construct systematic knowledge frameworks, accumulate tacit knowledge, and solidify the resource foundation for personal knowledge management ability. Simultaneously, their vibrant research environments stimulate pilots’ proactive knowledge management awareness, fostering an inertia for resource accumulation. This initial resource advantage reduces resource depletion during knowledge management, enhances resilience in adversity, and accelerates the conversion of personal knowledge management ability into career resilience. Consequently, pilots from key universities demonstrate a stronger promotion of career resilience through their personal knowledge management ability compared to those from non-key universities.

**Table 10:** Heterogeneity analysis results.

Variables	Key universities	Non-key universities	Domestic aviation schools	Foreign aviation schools
	Model 1	Model 2	Model 3	Model 4
*PKMA*	0.773*** (7.019)	0.636*** (14.788)	0.707*** (15.485)	0.513*** (6.600)
*AG*	−0.037 (−0.264)	−0.119* (−2.181)	−0.197** (−3.034)	0.010 (0.128)
*MS*	−0.085 (−1.064)	−0.055 (−1.824)	−0.050 (−1.434)	−0.078 (−1.635)
*HEA*	−0.524 (−1.651)	0.840* (2.378)	−0.572 (−1.971)	0.541 (1.378)
*CB*	0.082 (0.818)	0.075 (1.819)	0.072 (1.455)	0.073 (1.260)
*Constant*	1.408 (2.016)	−0.913 (−1.349)	1.841** (3.113)	−0.191 (−0.262)
*R* ^2^	0.696	0.593	0.638	0.555
∆*R*^2^	0.627	0.582	0.626	0.514
*F-Value*	10.095	51.391	50.830	13.482

*p < 0.05, **p < 0.01, ***p < 0.001. Values in parentheses represent *t*-values.

Models 3 and 4 in Table 10 show the results of the heterogeneity analysis of domestic and foreign aviation schools. It can be observed that, compared with Foreign Aviation Schools, the personal knowledge management ability of domestic aviation school pilots has a stronger promoting effect on career resilience. This difference is not accidental and needs to be analyzed from the perspective of the deep-seated mechanism of the relationship between knowledge management and career resilience in the training model. From the perspective of knowledge transmission models, domestic aviation schools generally adopt a distinctive training system combining “theoretical learning + mentor-based instruction.” In terms of theoretical learning, domestic aviation schools place greater emphasis on systematic study of foundational flight knowledge, aviation meteorology, civil aviation regulations, navigation, and aeronautical charts during instructional phases. Regarding mentor-based instruction, the apprenticeship system enables trainees to gradually absorb implicit expertise from instructors in flight skills and crisis management during routine flight training. This training model not only helps students build a comprehensive framework of basic knowledge, but also strengthens their ability to update, store, integrate, and apply knowledge. When students enter the career development stage, this systematic knowledge management ability can be more effectively transformed into resilience to cope with career challenges. In contrast, foreign aviation schools are influenced by market orientation, and their training models focus more on the efficiency of obtaining licenses. Their teaching content is mostly based on the license exam syllabus, with insufficient attention to the depth and systematic nature of knowledge. Knowledge reserves mostly remain at the level of “exam-oriented” explicit knowledge, lacking channels for the absorption of tacit knowledge. This model may lead to the fragmentation of students’ knowledge systems, making it difficult to form effective linkages and greatly reducing the efficiency of knowledge utilization, which in turn affects the building of career resilience. In summary, the differences between domestic and foreign aviation schools in their training models affect the systematic nature of knowledge transfer, the integrity of the knowledge system, and the degree of adaptation to industry requirements. Ultimately, the personal knowledge management ability of pilots trained at domestic aviation schools has a more significant effect on promoting career resilience.

## 5 Conclusion and discussion

### 5.1 Research conclusions

**Figure 3:**
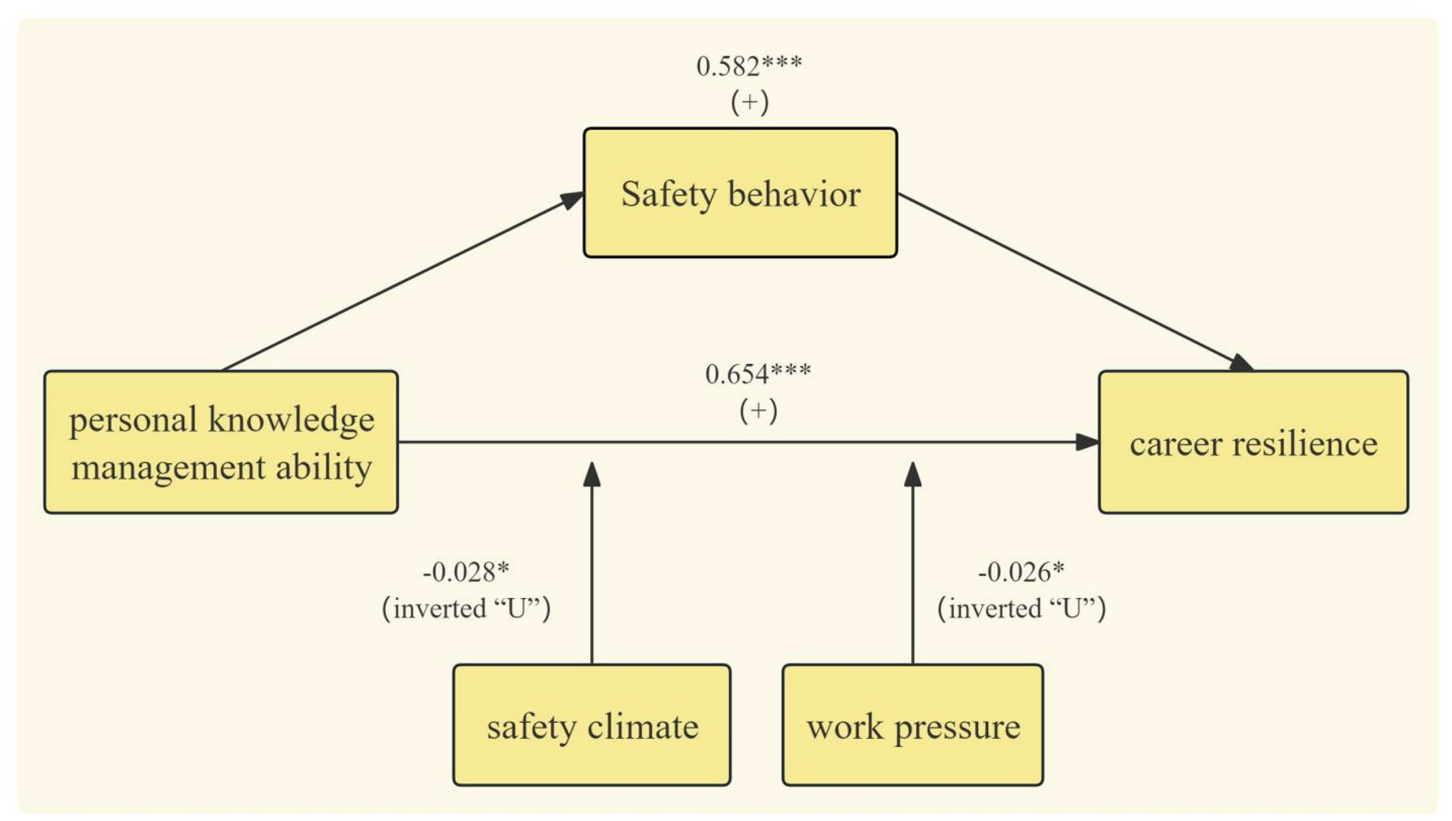
Results of regression, mediation, and moderation effects.

Through empirical analysis, this study mainly draws the following conclusions, as shown in Figure 3: First, personal knowledge management ability can significantly and positively influence the career resilience of civil aviation pilots; personal knowledge management ability and safety behavior had significant regression coefficients on career resilience, verifying the mediating role of safety behavior in both. Second, safety climate and work pressure show an inverted “U” shaped moderating effect in the process of personal knowledge management ability influencing the career resilience of civil aviation pilots. Third, heterogeneity analysis found that compared with non-key universities, the personal knowledge management ability of key university pilots has a stronger promoting effect on career resilience; compared with foreign aviation schools, the personal knowledge management ability of domestic aviation school pilots has a stronger promoting effect on career resilience.

Through the above conclusions, this study obtained the following research insights:

1. Enhance personal knowledge management ability and strengthen the link between knowledge effectiveness and career resilience.

From the perspective of knowledge management theory, improving personal knowledge management ability is the foundation for building career resilience in pilots. Deeply embedding knowledge management modules into the pilot training system can systematically enhance their cognitive flexibility and ability to cope with complex environments. Therefore, it is necessary to build a structured personal knowledge management framework based on the pilot skill lifecycle management system, including the three dimensions of technical operation, decision-making, and behavioral norms. At the technical operation level, typical deviation cases from Quick Access Recorders (QAR) data and emergency response checklists (e.g., single-engine failure, wind shear go-around procedures) should be integrated and stored with tags based on aircraft type, phase, and risk level. At the decision-making level, cockpit resource management (CRM) conflict cases and fatigue threshold data should be collected, and models linking physiological indicators (e.g., heart rate variability) to decision quality should be established. At the behavioral norms level, visualize the comparison between non-compliant operations (such as flying around thunderstorms without reporting) and compliant operations, and focus on the pilot’s operational and disposal capabilities at key behavioral nodes. By comprehensively consolidating the foundation of pilots’ personal knowledge and skills, it also provides strong support for the development of resilience.

1. Build an organizational support system to unleash the potential of career resilience capital.

According to organizational management theory, organizational support systems play an important role in cultivating employees’ career resilience capital. Airline management should systematically assess and optimize the safety climate and workload to ensure that they remain in the positive range of the inverted U-shaped curve. Invest heavily in the construction of an enterprise-level knowledge management system, integrate flight data, QAR analysis, safety reports, and best practice cases, and design incentive mechanisms (such as knowledge contribution points) to promote high-quality knowledge sharing and reuse. Incorporate knowledge contribution and application abilities into the pilot performance evaluation and promotion system, and deeply bind individual knowledge management abilities with the construction of organizational safety resilience capital.

1. Strengthen international cooperation to promote mutual assistance in pilot knowledge management from a global perspective.

Knowledge management plays a significant role in cross-organizational and cross-border knowledge transfer. Civil aviation institutions and airlines in different countries have distinct characteristics in pilot training and management. Strengthening international cooperation can facilitate knowledge sharing and exchange. For example, establishing an International Pilot Knowledge Management Alliance and regularly hosting global knowledge-sharing conferences encompassing three key areas—flight safety research, operational experience exchange, and training resource sharing—would enable pilots from various countries to share flight experiences, emergency response cases, and other knowledge, thereby broadening their horizons and enriching their knowledge reserves. At the same time, cross-cultural knowledge management research should be carried out to address differences in career resilience among pilots from different academic backgrounds, and targeted training programs should be developed, such as experience inheritance projects between key and non-key universities, and cooperation and exchange programs between domestic and foreign aviation schools. This will not only enhance the overall career resilience of pilots worldwide, but also strengthen the civil aviation industry’s ability to respond to complex international environments and global challenges, and promote the safety and development of the global civil aviation industry. This not only provides reference and inspiration for knowledge management among Chinese civil aviation pilots but also offers theoretical and practical guidance for knowledge management among global civil aviation pilots.

### 5.2 Research contributions

The main contributions of this study are as follows:

1. In terms of theoretical contributions, this study expands the research content of pilot knowledge management and career resilience, revealing the mediating chain mechanism between safe behavior, personal knowledge management ability, and career resilience. This study refines the internal logic of the transformation of knowledge management ability into career resilience, compensating for the shortcomings of existing research in its insufficient attention to complex process mechanisms. At the same time, previous research on knowledge management ability has mainly focused on the meso level and rarely touched on the relationship with career resilience. This study shifts the focus from the organizational level to the micro level, enriching the research content on personal knowledge management and career resilience in the field of civil aviation pilots.
2. In terms of practical contributions, the results of this study have important reference and learning significance for ensuring civil aviation flight safety. It provides a dual-track improvement path of “knowledge management + safe behavior” for pilot training, enabling personal knowledge management ability to be effectively transformed into career resilience through safe behavior, thereby strengthening flight safety defenses at the individual level. At the same time, this study provides a quantitative basis for airlines to optimize the safety climate and alleviate flight pressure. Although this study focuses on the practical context of Chinese civil aviation pilots, its findings offer important reference and insights for pilot training and management policies in the international civil aviation community.

### 5.3 Research limitations and future directions

The sample of Chinese civil aviation pilots has certain limitations. Specifically, the following four aspects have a certain impact on the research results:

1. This study only focuses on exploring the impact of personal knowledge management ability on the career resilience of civil aviation pilots. However, in reality, there are many factors that affect the career resilience of civil aviation pilots. These factors do not exist in isolation; they may be intertwined and interact with each other in the process of career resilience development and change. Therefore, the perspective of personal knowledge management ability has certain limitations and cannot comprehensively cover and deeply analyze all variables that may affect career resilience and their complex interaction mechanisms. At the same time, this study uses cross-sectional data and cannot observe the dynamic causal relationship between variables. In the future, tracking studies can be used to examine the evolutionary relationship between pilots’ knowledge management ability and career resilience in long-term flight missions.
2. Model complexity limits the statistical power of inference in small-to-medium sample sizes. In complex models, particularly those involving both mediating effects and nonlinear moderation effects, a sample size of 210 exhibits a certain mismatch with the complexity of the constructed model. This is especially evident when testing the curvilinear moderation effects of safety climate and work pressure, where the sample size provides limited support for statistical power. Concurrently, this study suffers from a limitation of gender imbalance in its sample composition. The absence of female participants may restrict the generalizability of findings to female pilots’ occupational characteristics. Future research should collect female pilot data to comprehensively explore potential gender-related influences on the variables under investigation and mitigate sample bias.
3. The sample of 210 Chinese civil aviation pilots used in this study reflects the “Three Foundations Construction” (grassroots, fundamentals, and basic skills) and Pilot Lifecycle Management (PLM) policies implemented by the Civil Aviation Administration of China (CAAC), which have shaped specific knowledge management behavior patterns among pilots. This deep integration enables the study’s conclusions to provide directly actionable recommendations for management practices in Chinese airlines. In particular, the inverted U-shaped inflection point between safety climate and work pressure can provide a strong basis for the formulation and optimization of pilot fatigue management policies, helping to improve pilot work efficiency and safety. However, the authoritarian safety leadership style of Chinese civil aviation and its reliance on CAAC supervision may make these conclusions inapplicable to pilots in non-high-compliance industries or under Western union systems. Pilot groups in different cultural and institutional environments may exhibit distinct behavioral patterns and coping mechanisms, and the study’s conclusions cannot fully reflect the occupational characteristics, cultural backgrounds, and work environment differences of pilots in other countries and regions. Therefore, caution is required when extrapolating the study’s conclusions.
4. The core contribution of this study is to reveal the existence of the mediating chain of “personal knowledge management ability—safety behavior—career resilience” and to propose the hypothesis of the inverted U-shaped moderating effect of safety climate and work pressure. This finding provides baseline evidence for the management and development of career resilience among Chinese civil aviation pilots and has important theoretical significance. It opens up new directions for follow-up research and promotes a deeper understanding of the formation mechanism of career resilience among pilots. However, due to the limitations of the sample size, the path coefficients (such as the mediating effect of safety behavior) and the location of the inverted U-shaped inflection point still need to be verified by follow-up large-sample studies. At present, this study should be positioned as an exploratory validation of the theoretical framework rather than a definitive conclusion. Despite its limitations, its exploratory value is undeniable, providing valuable foundational insights and references for future research, which will help further refine and deepen the relevant theoretical framework. Therefore, the generalizability and validity of the research conclusions should be interpreted with caution. Future studies should expand the sample scope to include more countries and regions to validate the applicability of these conclusions among global pilot populations.

## Data Availability

The datasets presented in this article are not readily available because research data are not shared for ethical reasons. Requests to access the datasets should be directed to lai2301301020@163.com.

## Footnotes

^1^The Chinese civil aviation pilots referred to in this study are pilots of civil aviation companies who have obtained at least a commercial pilot license (CPL).

^2^According to data from the Civil Aviation Administration of China (CAAC), as of the end of 2023, China primarily had 66 airlines, with the four major groups being China Southern Airlines, China Eastern Airlines, Hainan Airlines, and China Southern Airlines.
